# Role of exogenous putrescine in the status of energy, DNA damage, inflammation, and spermidine/spermine-n(1)- acetyltransferase in brain ischemia-reperfusion in rats

**DOI:** 10.22038/IJBMS.2022.63733.14046

**Published:** 2022-05

**Authors:** Nihal Cetin, Dervis Dasdelen, Rasim Mogulkoc, Esma Menevse, Abdulkerim Kasim Baltaci

**Affiliations:** 1 Department of Pharmacology, Faculty of Medicine, Selcuk University, Konya, Turkey; 2 Department of Physiology, Faculty of Medicine, Selcuk University, Konya, Turkey; 3 Department of Biochemistry, Faculty of Medicine, Selcuk University, Konya, Turkey

**Keywords:** 8-OHdG, Brain ischemia, IL-6, Nitric oxide, NF-kB, Putrescine, Rat, Spermidine/Spermine-N(1)

## Abstract

**Objective(s):**

This study aims to investigate the role of putrescine against brain ischemia-reperfusion (IR) injured rats administered with 250 µmol/kg exogenous putrescine and highlight the IR-associated mechanisms in energy metabolism and inflammatory pathway.

**Materials and Methods:**

The rats were divided into six groups: 1-Sham group; 2-IR group, 30 min of ischemia and 30 min of reperfusion was performed with bilateral carotid occlusion (BCAO); 3-IPR group, a single oral dose of putrescine was administered at the start of the 30-minute reperfusion; while in the other treatment groups, 4 doses of putrescine were given within 12-hour intervals. After 30 min of reperfusion, the first dose was administered immediately in the IR-PI (group 4), after 3 hr in IR-PII (group 5), and after 6 hr in IR-PIII (group 6). Interleukin-6 (IL-6), Nuclear factor NF-kappa-B (NF-kB), Adenosine triphosphate (ATP), total Nitric oxide (NO), 8-hydroxyguanosine (8-OHdG), Spermidine/Spermin N-acetyltransferase (SSAT) levels were analyzed in brain tissues.

**Results:**

IR reduced brain ATP levels; however, putrescine treatment reversed this state. Brain NO and 8-OHdG levels, and NF-kB and IL-6 levels increased significantly in the IR group and these elevations were decreased in putrescine administered groups. SSAT levels were higher in the IR-PII group. The lowest levels were observed in the IR-PIII group.

**Conclusion:**

The exogenous putrescine supplementation after cerebral IR creates neuroprotective effects independent of the time of administration; according to conditions such as formation of radicals in the brain, the spread of the inflammation and the need for consumption of energy are considered as a whole.

## Introduction

Ischemia is a condition resulting from decreased arterial or venous blood flow with an insufficient blood supply to the tissues and cell death (1, 2). Ischemia causes depletion of energy sources, especially ATP which is essential for cellular homeostasis and ultimately leads to ion imbalance in the cell membrane. Disturbance of the balance of Na^+^ and Ca^+2^ ions may cause especially acidosis or osmotic shock (3, 4). Cerebral ischemia is the restriction of blood flow in the brain (5) and causes depletion of oxygen and nutrients, and subsequently irreversible damage to cell functions in brain tissue (5). Even if oxygen levels are reduced in ischemic conditions, the damage may not be severe. However, when oxygen levels return to normal levels during reperfusion, an excess of hydrogen peroxide (H_2_O_2_) and superoxide radical (O_2_·-) is formed in the ischemic zone in which Xanthine oxidase (XO) plays a role. XO is an important enzyme that catalyzes conversion of O_2_ to superoxide radical and converts hypoxanthine to xanthine and subsequently to uric acid throughout reperfusion. Therefore, the formation of superoxide radicals causes damage (1). So, it is of vital importance to protect the tissue exposed to ischemia at the highest level from oxidative damage that may occur during reperfusion.

Polyamines are aliphatic molecules containing 2, 3, or 4 amino groups and are commonly found in nature and living organisms, almost all eukaryotes, and prokaryotes (6). Putrescine, spermidine, and spermine are known as the most important polyamines found in the brain. They are known to play important roles in cell growth and regulation of the cell membrane dynamics (including neurons) in mammals (7). Spermidine/Spermin N1-acetyltransferase (SSAT) is one of the most crucial enzymes in polyamine metabolism (8). The acetyl group was transferred to the aminopropyl groups of spermidine and spermine by SSAT (9). The increase in SSAT may contribute to elevation of putrescine and reduction in spermidine levels which occur after ischemia (10). Evidence from the present literature suggests that there is a strong relationship between high SSAT and reduced ATP levels in the metabolic process (9). It is also known that SSAT plays a key role in alterations of acetyl coenzyme A and ATP levels and the process of oxidative stress (11). Overproduced free oxygen radicals can react with macromolecules in cells and cause severe cellular damage, including lipid peroxidation, oxidative modification of proteins, and DNA oxidation. 8-hydroxy guanosine (8-OHdG) is a biomarker of this oxidative DNA damage (12). Studies on the interaction of nitric oxide (NO) with oxygen radicals and its antioxidant properties have shown controversial results (13). High NO concentrations have been reported in studies of cerebral ischemia-reperfusion (13, 14). It is evidenced that neuronal damage occurs via NF-kB pathway’s activation and SSAT-induced polyamine oxidation (8). 

Neurological studies show that brain polyamines associate with neurodegenerative disorders. Pharmacological and transgenic animal studies indicate that reduced polyamines increase the expression of cortical neuron Nicotinic acetylcholine receptor (nAChR) and manage nicotine receptor-mediated neuroprotection (15).

NF-kB, which is an important immune and anti-inflammatory response regulator, can be activated by pathogens and inflammatory mediators via the p65:p50 heterodimer. However, cytokines and chemokines are also important biomarkers utilized to determine the severity of drug-induced liver injury (16). 

Briefly, brain tissue damage can cause irreversible losses. It is not always possible for patients in this situation to attempt immediately for treatment. Therefore, we hoped to reveal promising results in brain ischemia by administering exogenous putrescine in terms of inflammation, energy use, and DNA damage, depending on different application times. Considering the above-mentioned facts, we sought to elucidate the cellular and molecular mechanisms underlying the co-regulation of SSAT, IL-6, NF-kB, ATP, NO, and 8-OHdG levels involved in cerebral IR in the present study. Another aim of this study was to evaluate the potential effects of putrescine applied at different time intervals following the cerebral IR procedure. 

## Materials and Methods

This study was carried out using 46 male albino Wistar rats weighing 200–250 grams, which were obtained from Selcuk University Experimental Medicine Research and Application Center. The study was approved in the decision of Selcuk University Experimental Medicine Application and Research Center Ethics Committee (no: 2019-27, date: 28.06.2019). It was supported by Selcuk University Scientific Research Projects Coordinatorship with project number 20401001.

***Surgical procedure and experimental groups***

In rats anesthetized by intraperitoneal administration of ketamine HCl (60 mg/kg) and xylazine (5 mg/kg), the carotid arteries were carefully isolated after making a ventral incision at the midline of the neck. brain ischemia occurred by making occlusion in the carotids. Both carotid arteries were isolated using the method used by Oz *et al*. (17). In the putrescine treatment group 250 µmol/kg of putrescine was applied by gavage (18, 19). Putrescine administration times have been planned considering the half-life of SSAT. 

Sham group (n=6): After isolation of the carotid arteries from surrounding tissues, intracardiac blood was collected from animals under general anesthesia. Then cervical dislocation was performed.

Ischemia-Reperfusion Group (I/R) (n=8): Carotid arteries were ligated for 30 min to induce ischemia. Afterward, reperfusion was achieved for 30 min. After reperfusion, cervical dislocation was performed.

Ischemia + Putrescine + Reperfusion Group (IPR) (n=8): Carotid arteries were ligated for 30 min to induce ischemia followed by 250 µmol/kg putrescine administration by gavage. Subsequently, animals were reperfused for 30 min and then cervical dislocation was performed.

Ischemia-Reperfusion + Putrescine Group (I/R-PI) (n=8): Carotid arteries were ligated for 30 min to induce ischemia and then reperfusion was induced for 30 min, followed by 250 µmol/kg putrescine administration by gavage. Additional doses were administered at 12-hour intervals. Tissue samples were taken 12 hr after the final dose (48 hr after IR), then cervical dislocation was performed.

Ischemia-Reperfusion + Putrescine Group (I/R-PII) (n=8): Carotid arteries were ligated for 30 min to induce ischemia and then reperfusion was induced for 30 min. 250 µmol/kg putrescine administration was performed by gavage after 3 hr of IR. Three more doses were administered at 12 hr intervals. Tissue samples were taken 12 hr after the final dose of putrescine (51^st^ hour following IR) and cervical dislocation was performed.

**Figure 1:**
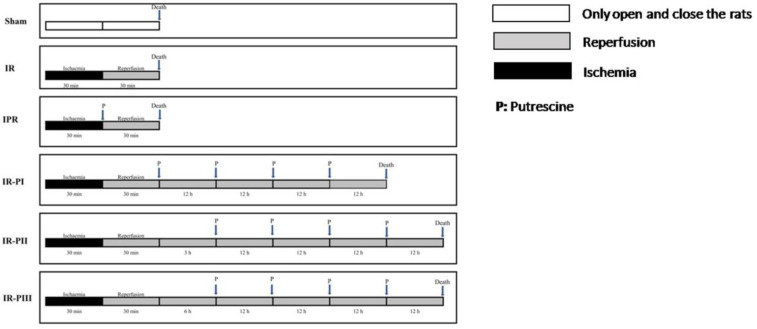
Procedure of the surgical and putrescine application in experimental groups

 Ischemia-Reperfusion + Putrescine Group (I/R-PIII) (n=8): Carotid arteries were ligated for 30 min to induce ischemia and then reperfusion was induced for 30 min. 250 µmol/kg putrescine administration was followed by gavage after 6 hr. Three more doses were administered at 12-hour intervals. Tissue samples were taken 12 hr after the final dose of putrescine was administered (54^th^ hour following IR) (Figure 1).

***Experimental procedure***

At the end of the experiment, the rats in all groups were sacrificed by cervical dislocation under general anesthesia. After weighing the brain tissues, they were placed in a homogenizer (Misonix Microscan ultrasonic tissue shredder) and homogenized at 4 °C. Samples were centrifuged at 3000 rpm for 15 min and supernatants were stored at -80 °C until analysis. 

***Preparing the putrescine for oral application***

Putrescine dihydrochloride (catalog no: Sigma P7505) was dissolved in distilled water and one dose was adjusted to be 250 µl/kg rat. Putrescine administration was done by gavage by adding saline as much as the solution volume of putrescine.

***Biochemical analysis***

SSAT, IL-6, NF-kB, ATP, NO, and 8-OHdG were analyzed in brain tissue samples. The analyses were carried out in Selcuk University Medical Faculty Physiology Department Research Laboratories. Measurements were done with ELISA and spectrophotometric methods. The experiments were performed by using the ELISA reader BMG LABTECH (Germany) in accordance with the manufacturer’s instructions.

The commercial test kits used in the experiments were as follows; IL-6 (BT-Lab, catalog no: E0135Ra) (ng/g tissue), Nf-kB (BT-Lab, catalog no: E0287Ra) (ng/g tissue), ATP (BT-Lab, catalog no: E0920Ra) (ng/g tissue), NO (Cayman, catalog no:780001) (µM/g tissue), the SSAT (BT-Lab, catalog no: E2456Ra), (ng/g tissue), and 8-OHdG (BT-Lab, catalog no: E0031Ra) (ng/g tissue).

***Statistical analysis***

The statistical evaluation of the findings was performed using the IBM SPSS 22.0 (Armonk, NY: IBM Corp.) package program, and the means and standard deviations of all parameters were calculated. In order to determine the homogeneity of the data. The Levene test was used, and the Shapiro-Wilk test was performed for confirming the normality assumption for the data. One-way analysis of variance (ANOVA) test was used to determine the differences between the groups, and the Tukey test which is one of the *post-hoc* tests, was used to determine in which group the difference originated. The difference at the *P*<0.05 level was considered significant. Data is represented by mean ± SD. For all statistical tests, a value of *P*<0.05 was considered statistically significant.

## Results

***Results of total nitrate + nitrite (NO)***

The results of NO have been shown in Table 1. The highest NO levels in the brain tissue were found in the IR group, and the decreases were statistically significant in all putrescine-administered groups.

***Results of ATP***

When the level of ATP in the brain tissue was evaluated, the highest level was determined in the sham group, and the difference with the IR group was statistically significant. Supplementations of putrescine in different time periods showed an increase in the levels of ATP and this increase was significant compared with the IR group (Table 2).

***Results of SSAT***

When data shown in Table 3 were evaluated, it was determined that the results of the IR group were different from IPR and IR-PIII groups.

***NF-KB results***

**Figure 2:**
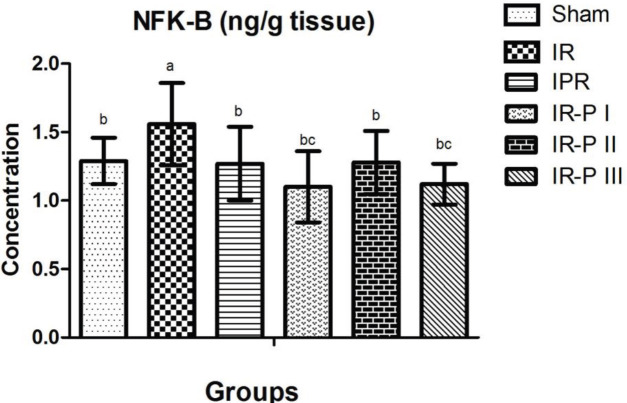
Comparison of brain NF-kB levels of the study groups a, b, c: Values with different superscript letters are significantly different from each other. A value of *P*<0.05 was considered statistically significant

As shown in Figure 2, NF-kB levels in brain tissue were significantly higher in the IR group than in the sham group. In the putrescine supplemented groups, the NF-kB levels were significantly reduced compared with the IR group.

***IL-6 results***

**Figure 3:**
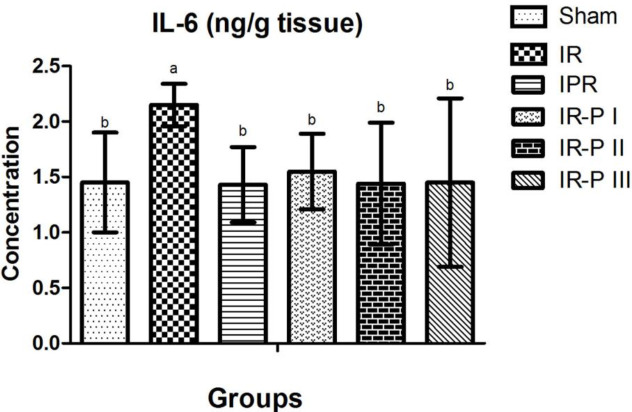
Comparison of brain IL-6 levels of the study groups a, b: Values with different superscript letters are significantly different from each other. A value of *P*<0.05 was considered statistically significant

Measurements of IL-6 levels in brain tissue showed the highest levels in the IR group and these values were statistically significant when compared with the sham group. IL-6 levels in the putrescine supplemented groups were found to be decreased, and this decrease was found to be statistically significantly different when compared with the IR group (Figure 3).

***The results of 8-OHdG***

Findings of 8-OHdG, are shown in Table 4, and the highest levels were found in the IR group brain tissue. In the putrescine supplemented groups, DNA damage was decreased in the brain tissue. 

## Discussion

Brain ischemia-reperfusion can cause serious and irreversible health problems. There is not much information on ischemia-reperfusion reported by previous studies about the effects of putrescine which is one of the important polyamines. In the present study, the metabolic effects of putrescine supplementation in IPR, I/R-PI, I/R-PII, and I/R-PIII experimental groups were investigated comprehensively.

It is known that reactive oxygen species are involved in ischemia-reperfusion injury in most tissues. In this study, the highest NO levels in brain tissue were found in the IR group, and the decrease in all putrescine supplemented groups was found statistically significant. These results indicate that exogenous putrescine administration has a time-independent effect on IR, and there is no difference between administering immediately after reperfusion and administering 3 and 6 hr after IR. Putrescine administration played a significant role in the decrease of NO values, which we expressed as total nitrate/nitrite. In this sense, the increase in oxygen radical production, which is one of the damages caused by IR, was prevented by supplementation of putrescine. The decrease in the levels of NO3-(nitrate), which is formed as a result of the forward reaction of superoxide oxygen radicals and ONO2-(nitrite), shows that radical formation is reduced and this situation occurs with the application of putrescine. In cerebral ischemia-reperfusion studies, high NO concentrations were determined in the brain (13, 14, 20). Although different agents with neuroprotective properties are used in these studies, our data are consistent with the findings of the previous studies. Our study reveals the NO lowering effects of putrescine and thereby suggests a relationship between the supplementation of putrescine and decrease in the formation of free radicals. XO uses the oxygen that becomes available to the cells upon reperfusion as an oxidant and converts hypoxanthine to xanthine and eventually to the uric acid. In the last two steps, superoxide anion (O2 •−) is formed from reactive oxygen species. O2 •− formed under normal physiological conditions is first converted to hydrogen peroxide by superoxide dismutase (SOD), and then to water by catalase. However, increasing O2 •− and hydrogen peroxide (H_2_O_2_) due to the effect of XO in IR exceeds the antioxidant capacity and cannot be effectively removed from the cellular environment (21).

When the ATP levels in the brain tissue were evaluated, the highest level was determined in the sham group, and the difference between IR and sham groups was statistically significant. Exogenous putrescine applied at different time points caused an increase in ATP concentrations. When this increase in brain tissue was compared with the IR group, it was found to be statistically significant in the I/R-PIII group. In the brain tissue, the ATP level in the I/R-PIII group was found significantly higher than in the IR group. This finding shows that ATP production in the brain increases with the addition of exogenous putrescine. This also indicates that application of putrescine especially 6 hr after reperfusion is more effective in regulation of the energy rather than early stages of supplementation.

The SSAT levels in the IR group significantly differ from IPR and IR- PIII groups. Acetyl polyamines are important markers for increased SSAT activity in the cells, and their accumulation is related to their degradation by acetyl polyamine oxidase (APOA). Additionally, transcription and translation processes increase in the presence of high concentrations of polyamines and polyamine analogs while degradation of SSAT protein decreases (9). Furthermore, evidence suggests that there is a direct relationship between high SSAT levels and a significant decrease in ATP levels in the metabolic process (9). Our findings support the relationship between ATP and SSAT levels reported by other study groups. While SSAT levels were high in our IR groups, ATP levels were low. It has been confirmed that many cells require SSAT for conversion of spermine and spermidine to putrescine. In the absence of SSAT, the conversion of spermin to spermidine in small amounts is possibly the result of direct oxidation of spermine by spermine oxidase enzyme (SMO) (22). Interestingly, it shows that putrescine plays a more critical role than spermidine in promoting neoplastic growth. Even though putrescine is thought to be the precursor of spermidine and spermine, it also influences gene regulation either by its own actions or by modulating the effects of these amines. This state is the SSAT/APAO pathway, which includes putrescine, reactive oxygen species, and N-acetylaminopropanal, and rises to notice. Therefore, putrescine plays a critical role in the SSAT/APAO pathway that gives rise to oxidative damage (9). As a matter of fact, SSAT levels in the putrescine supplemented groups in our study were decreased except for IR-PII, similar to those found in the literature as reported by Babu *et al*. (18). In their study, which evaluated the role of SSAT after transient cerebral ischemia and reperfusion, they described a slight but significant increase 12 hr after reperfusion following ischemia (18). They argued that this situation does not contribute much to the production of putrescine by the back-conversion pathway in cortex SSAT activity. However, they showed that in the striatum, polyamine interconversion is more active at 9–18 hr after 2 hr of ischemia-reperfusion, which is reflected by a sustained increase in SSAT activity (18). Therefore, regulation of SSAT in the brain is impaired in IR injury (10, 18). It has been reported that the deficiency of polyamines also contributes to organ damage after IR (18). The levels of SSAT for IR-PII and IR-PI compared with sham and IR groups considered that pool of catabolism and secretion of intracellular polyamines did not show any relations. But, compared with the sham, IR group with the IR-PIII group (*P*<0.05) we conclude that putrescine administration is important for SSAT activity via time interval-dependent. Therefore, we also conclude that exogenous putrescine administration after 0–3 hr and 30 min ischemia-reperfusion is more active than at 6 hr. As seen in Table 3, the IR-PIII group has the lowest SSAT levels, and we infer that the time of putrescine administration after IR affects the activity of SSAT. But, comparing with the sham, IR and the IR-PIII groups (*P*<0.05), we conclude that putrescine administration is important for SSAT activity via time interval-dependent.

There are also studies showing that polyamines cause serious damage in brain ischemia-reperfusion by disruption of the blood-brain barrier (23). It has been reported that the polyamine conversion pathway has an important role in the post-ischemic increase of putrescine levels, and it can be protective against neuronal cell damage after transient cerebral ischemia by blocking this pathway (24).

On the other hand, NF-kB levels in the brain were found significantly higher in the IR group than in the sham group. It was determined that there was a significant decrease in the NF-kB values measured in the brain tissue in all putrescine supplemented groups compared with the IR group. NF-kB, an important immune and anti-inflammatory response regulator is an additional transcription factor used to determine the severity of drug-induced oxidative damage (8). Based on these considerations, NF-kB levels were assessed to determine whether there was tissue damage induced by addition of putrescine in this study. Our results showed that putrescine did not cause tissue damage, and NF-kB analysis showed similar results in putrescine applications given in different time manners. Our results appear to be in agreement with the result of Liu *et al* (8). They discussed that the mechanism underlying the role of acrolein in stroke-related neuronal damage is activation of the SSAT-induced polyamine oxidation-induced NF-kB pathway. These results provide a new mechanism of neurotoxicity in stroke patients, contribute to the development of neutralizing or preventive precautions, and insight into our aspects of neural protection (8). In recent years, studies have not been possible to fully elucidate the precise mechanisms of inflammatory responses following ischemic stroke since inflammation is a complex mechanism of interactions between inflammatory cells and molecules. Therefore, in studies conducted using several different agents, it has been suggested that modulation of neuroinflammation and inflammatory signaling pathways in brain ischemia is important. So that we can postulate that these findings comprehend potential targets for ischemic stroke treatment.

The role of IL-6 in ischemic stroke has been clarified less than other well-defined cytokines in the previous studies. When we evaluated the IL-6 data, we found the highest levels in the IR group, and the difference between these values and the results from the sham group was statistically significant. IL-6 levels showed a significant decrease in all groups in which putrescine was supplemented. Regarding our IL-6 level results in which we observed reduction in the brain tissue in sham and putrescine administered groups, the difference was significant compared with the IR group. These findings also supported that putrescine application did not cause tissue damage. It has been reported (25) that expression of IL-6 continues to increase up to 24 hr from the beginning of ischemia (25). In line with our results, Okumura *et al*. (19), studying the effect of orally administered polyamines in liver ischemia, reported a significantly decreased IL-6 level in the polyamine group. However, it was evidenced that IL-6 receptor antagonist-treated or IL-6-deficient mice did not differ from untreated mice and IL-6 was not actively involved in the pathogenesis of ischemia (26, 27). There are also publications reporting that IL-6 is important in ischemia (28).

8-OH/dG is a biomarker of this oxidative DNA damage (12). When our data in Table 4 were evaluated, oxidative DNA damage was found lower in the putrescine supplemented groups compared with the sham and IR groups. When compared brain tissue with the IR group, a statistically significant difference in 8-OH/dG levels was found in all putrescine-administered groups. Putrescine administration reduced the damage caused by IR in the brain tissue. It is thought that when NO and superoxide react, it directly causes DNA damage by formation of peroxynitrite. Thus, genetic deletion or pharmacological inhibition of iNOS leads to improved neurological outcomes (25). Studies on NO interacting with oxygen radicals and its antioxidant properties have yielded different results (13). High NO concentrations were determined in cerebral ischemia-reperfusion studies (13, 14). Dasdelen *et al*. (29) reported that whereas 8-OHdG levels increased significantly in I/R, these levels were significantly suppressed and DNA damage was prevented by 3’,4’-Dihydroxyflavonol (DiOHF) supplementation. DNA damage occurs through two pathways, endonuclease-mediated active DNA damage, and ROS-induced endonuclease-independent passive DNA damage. A significant increase in ROS caused by ischemia triggers premortality signaling in neurons by induction of apurinic/apyrimidinic regions in addition to base modifications, single-strand breaks, or double-strand breaks (2). Nowadays, even though there is no study about the analysis of the mentioned biochemical parameters in putrescine-applied brain ischemia-reperfusion models, we compared our study data with the findings of other researchers that studied different various agents that have antioxidant, anti-inflammatory, etc. properties.

**Table 1:** Brain Nitric oxide (NO) levels of the study groups

Groups	N	NO (µM/g tissue)	
Sham	6	1,46 ± 0,26 ab	
IR	8	1,73 ± 0,69 a	
IPR	8	1,08 ± 0,22 b	
IR-PI	8	1,30 ± 0,46 b	
IR-PII	8	1,31 ± 0,17 b	
IR-PIII	8	1,25 ± 0,30 b	

Compared with all other study groups, brain NO levels were increased in the IR group. The Tukey test is used. a, b: Values with different superscript letters are significantly different from each other. A value of *P*<0.05 was considered statistically significant

**Table 2:** Brain adenosine triphosphate (ATP) levels of the study groups

Groups	N	ATP (ng/g tissue)
Sham	6	103,40 ± 17,36 a
IR	8	84,76 ± 2,56 b
IPR	8	95,44 ± 5,77 ab
IP-PI	8	89,15 ± 10,19 b
IR-PII	8	95,69 ± 10,59 ab
IR-PIII	8	102,72 ± 7,53 a

Compared with all other study groups, brain ATP levels were found lower in the IR group. The Tukey test is used. a, b: Values with different superscript letters are significantly different from each other. A value of *P*<0.05 was considered statistically significant

**Table 3:** Brain Spermidine/Spermin N-acetyltransferase (SSAT) levels of the study groups

Groups	N	SSAT (ng/g tissue)
Sham	6	0,53 ± 0,054 ab
IR	8	0,62 ± 0,11 a
IPR	8	0,50 ± 0,06 b
IR-PI	8	0,60 ± 0,17 ab
IR-PII	8	0,68 ± 0,12 a
IR-PIII	8	0,47 ± 0,12 b

Brain SSAT levels were reduced in IPR and IR-PIII groups when compared with the IR group. The Tukey test is used. a, b: Values with different superscript letters are significantly different from each other. A value of *P*<0.05 was considered statistically significant

**Table 4:** Brain 8-OH/Gua levels of the study groups

**Groups**	**N**	**8-OH/Gua** **(ng/g tissue)**
**Sham**	**6**	0,68 ± 0,18 ^b^
**IR**	**8**	1,38 ± 0,40 ^a^
**IPR**	**8**	0,66 ± 0,13 ^b^
**IR** **-PI**	**8**	0,63 ± 0,26 ^b^
**IR** **-PII**	**8**	0,63 ± 0,25 ^b^
**IR** **-PIII**	**8**	0,54 ± 0,20 ^b^

## Conclusion

These results show that putrescine supplementation has an independent from the time of application effect. Even in the group supplemented with putrescine inflammation was prevented and the tissues were protected from radical damages. In addition, putrescine supplementation contributed to the supply of energy. However, it is clear that the time of putrescine administration after IR affects the activity of SSAT. IL-6 and anti-inflammatory response regulator (NF-kB) levels were evaluated, and it was determined that inflammation was significantly reduced in all putrescine supplemented groups, and putrescine contributed to the anti-inflammatory response.

## Authors’ contributions

NC Performed conceptualization, investigation, methodology, visualization, writing, review, editing, and supervision. DD Helped with data curation, methodology, formal analysis, and software. RM Helped with methodology, investigation, software, writing, review, and editing. EM Performed investigation, conceptualization, data curation, writing, review, and editing. AKB Provided methodology, investigation, and formal analysis.

## Conflicts of Interest

The authors declare that they do not have any conflicts of interest.
